# Existence of SARS-Cov-2 in the Peritoneal Fluid

**DOI:** 10.1055/s-0043-1770129

**Published:** 2023-06-20

**Authors:** Orkun Ilgen, Mehmet Eyuphan Ozgozen, Ozgur Appak, Begum Ertan, Hikmet Tunc Tımur, Omer Erbil Dogan, Cemal Posacı

**Affiliations:** 1 Gynecologic Oncology Clinic, Erzurum City Hospital, Erzurum, Turkey; 2 Department of Obstetrics and Gynecology, Dokuz Eylul University School of Medicine, Izmir, Turkey; 3 Department of Microbiology, Dokuz Eylul University School of Medicine, Izmir, Turkey; 4 Obstetrics and Gynecology Clinic, Sandikli State Hospital, Afyon, Turkey; 5 Obstetrics and Gynecology Clinic, Urla State Hospital, Izmir, Turkey

**Keywords:** SARS-CoV-2, peritoneal fluid, surgical smoke, amniotic fluid, COVID-19, SARS-CoV-2, fluido peritoneal, fumaça cirúrgica, líquido amniótico, COVID-19

## Abstract

**Objective** To determine the existence of SARS-CoV-2 in the peritoneal fluid to assess the risk of exposure through surgical smoke and aerosolization threatening healthcare workers during abdominal surgery.

**Background** SARS-CoV-2 is a respiratory virus and possible ways of viral transmission are respiratory droplets, close contact, and fecal-oral route. Surgeries pose risk for healthcare workers due to the close contact with patients. Aerosolized particles may be inhaled via the leaked CO _2_ during laparoscopic procedures and surgical smoke produced by electrocautery.

**Methods** All the data of 8 patients, who were tested positive for COVID–19, were collected between August 31, 2020 and April 30, 2021. Recorded clinicopathologic data included age, symptoms, radiological and laboratory findings, antiviral treatment before surgery, type of surgery and existence of the virus in the peritoneal fluid. Nasopharyngeal swab RT-PCR was used for the diagnosis. COVID–19 existence in the peritoneal fluid was determined by RT-PCR test as well.

**Results** All 8 COVID–19 positive patients were pregnant, and surgeries were cesarean sections. 1 of the 8 patients was febrile during surgery. Also only 1 patient had pulmonary radiological findings specifically indicating COVID-19 infection. Laboratory findings were as follows: 4 of 8 had lymphopenia and all had elevated D-dimer levels. Peritoneal and amniotic fluid samples of all patients were negative for SARS-CoV-2.

**Conclusion** SARS-CoV-2 exposure due to aerosolization or surgical fumes does not seem to be likely, provided the necessary precautions are taken.

## Resumo

**Objetivo** Determinar a existência de SARS-CoV-2 no fluido peritoneal para avaliar o risco de exposição através da fumaça cirúrgica e aerossolização que ameaçam os profissionais de saúde durante a cirurgia abdominal.

**Contexto** O SARS-CoV-2 é um vírus respiratório e as possíveis formas de transmissão viral são gotículas respiratórias, contato próximo e rota fecal-oral. As cirurgias representam risco para os profissionais de saúde devido ao contato próximo com os pacientes. As partículas aerossolizadas podem ser inaladas através do CO2 vazado durante os procedimentos laparoscópicos e a fumaça cirúrgica produzida pela eletrocauterização.

**Métodos** Todos os dados de 8 pacientes, que foram testados positivos para COVID-19, foram coletados entre 31 de agosto de 2020 e 30 de abril de 2021. Dados clinicopatológicos registrados incluíam idade, sintomas, achados radiológicos e laboratoriais, tratamento antiviral antes da cirurgia, tipo de cirurgia e existência do vírus no fluido peritoneal. O diagnóstico foi feito através do swab nasofaríngeo RT-PCR. A existência de COVID-19 no fluido peritoneal foi determinada pelo teste de RT-PCR também.

**Resultados** Todas as 8 pacientes positivas para COVID-19 estavam grávidas, e as cirurgias eram cesarianas. 1 das 8 pacientes estava com febre durante a cirurgia. Também apenas 1 paciente tinha achados radiológicos pulmonares especificamente indicando infecção por COVID-19. Os achados laboratoriais foram os seguintes: 4 de 8 tinham linfopenia e todas apresentavam níveis elevados de D-dímero. Amostras de fluido peritoneal e líquido amniótico de todas as pacientes foram negativas para SARS-CoV-2.

**Conclusão** A exposição ao SARS-CoV-2 devido à aerossolização ou fumaças cirúrgicas não parece ser provável, desde que sejam tomadas as precauções necessárias.

## Introduction

Severe acute respiratory syndrome coronavirus 2 (SARS-CoV-2) outbreak was declared as a pandemic by the World Health Organization (WHO) on January 30, 2020. 1 The disease initially appeared in China and spread to many other countries rapidly. Human to human transmission was demonstrated shortly after the outbreak and the virus infected millions of people around the world in the following months. Early reports showed the mortality rate as 2.3% in China while as %1.6 in other countries. 2

SARS-CoV-2 is a respiratory virus and possible ways of viral transmission are respiratory droplets, close contact, and fecal-oral route. 3 Operating rooms (OR) are among risky places in situations like viral outbreaks because patients and healthcare workers are often in close contact. National and international surgical guidelines still evolve to protect healthcare workers from the risks posed by their colleagues and patients.

Inhalation of aerosolized particles is considered as a risk factor for transmission of SARS-CoV-2. 4 5 Surgical smoke due to electrocautery or the leaked CO2 that is used during laparoscopy may be inhaled in the OR. Mintz et al. claimed that laparoscopy should be used if the procedure is more suitable for the patient. They also stated that more evidence – based research is needed to determine the safety of laparoscopy. 5 Viral load of the peritoneal fluid seems to be the main factor that increases the risk of transmission through inhalation of leaked laparoscopic gases and smoke due to electrocautery.

This study was designed to evaluate patients, who were tested positive for COVID-19 and underwent obstetrical surgery, regarding the presence of viral genome in the peritoneal cavity.

## Methods

All women, who underwent surgery and had positive COVID-19 test results, at Dokuz Eylul University Hospital Department of Obstetrics and Gynecology between August 31,2020 and April 30, 2021 were included in the study. The diagnosis was determined with nasopharyngeal swab test. A positive test was defined as a positive result for SARS-CoV-2 with reverse transcriptase – polymerase chain reaction (RT-PCR) assay. Ethical approval for the study was obtained from the local ethics committee of Dokuz Eylul University Hospital (No:2021/04–41). Written informed consent forms were obtained from all participants.

The study was designed as a prospective study. The hospital electronic patient database provided epidemiological, clinical, laboratory and radiological data of the patients. Nasopharyngeal swab specimens were tested for SARS-CoV-2 at the microbiology laboratory of the same institution. The test was conducted by using virus mini kit (EZ1 Virus Mini Kit v2.0, Germany), followed by RT-PCR according to WHO guidelines.

Birth weight and gestational age at the delivery were recorded. Amniotic fluid samples were collected in addition to peritoneal irrigation fluid for the pregnant patients during the cesarean section (C/S). Peritoneal cavity was irrigated with 10 mL of saline solution and then the irrigation fluid was aspirated for testing. Peritoneal washing aspirate was also used by other researchers. 6 7 The peritoneal fluid sample was obtained before hysterotomy to prevent contamination. Testing for COVID-19 was performed 24/7 in the institution and therefore specimens were transferred to the laboratory momentarily in sterile vials provided by the laboratory. All biological samples were tested for SARS-CoV-2 with the same kit that was used for the nasopharyngeal specimens followed by RT-PCR according to WHO guidelines.

All analyses were performed by using IBM SPSS Statistics Version 25. Only descriptive statistics were calculated and given. Mean ± standard deviation was used to present the data.

This study was performed in consensus with our universitýs ethics guidelines. The ethics committee approval was obtained for this study (No:2021/04–41).

## Results

The mean age of patients was 28 ± 2 years. All patients, who underwent surgery when they were tested positive for COVID-19, were pregnant and all surgeries were cesarean sections. 3 of 8 (37.5%) patient were in their first pregnancy. All patients were at term during birth and only one of them was symptomatic while the other 7 were clinically asymptomatic. The symptomatic patient's body temperature was 39C during admission. This patient was operated 4 days after the onset of fever. Due to pregnancy, ionizing radiation was avoided and no patient had radiological imaging except obstetrical ultrasonography preoperatively. However, computerized tomography (CT) scanning was performed on one patient after birth (case 6). Ground – glass opacities and patchy lung consolidations were detected in the CT scan. None of the patients received antiviral medication preoperatively ( Tables 1 and 2 ).

**Table 1:** Characteristics of the patients

Case No.	Nasal swab for Covid-19	Age (year)	Parity	Gestational age (week)	Birth Weight (g)
1	+	26	0	39	3115
2	+	27	0	40	3348
3	+	30	1	37	3400
4	+	33	2	38	3600
5	+	30	1	39	3217
6	+	27	0	39	3300
7	+	26	2	39	3320
8	+	27	1	40	2960
Mean ± SD		28 ± 2			3282 ± 191

**Table 2:** Clinicopathologic data of the patients

Case No.	Fever	Cough	Dyspnea	Radiological finding	Time between symptoms and delivery (day)	Antiviral treatment before delivery	Covid-19 test in amniotic fluid	Covid-19 test in peritoneal fluid
1	−	−	+	−	4	−	−	−
2	−	−	- -	0	−		−	−
3	−	−	- -	0	−		−	−
4	−	−	- -	0	−		−	−
5	−	−	- -	0	−		−	−
6 7 8	- - -	- - -	- + - - - -	0 0 0	- - -		- - -	- - -

Blood samples from all patients were tested within 24 hours before surgery. Complete blood count, serum c – reactive protein (CRP), alanine aminotransferase, aspartate aminotransferase, blood urea nitrogen, creatinine concentrations and plasma d – dimer concentrations were recorded. Lymphopenia was detected in 4 of 8 patients and plasma d – dimer concentration was high in all 8 patients as laboratory findings related with COVID – 19 infection ( Table 3 ).

**Table 3:** Laboratory findings of the patients

Case No.	Leukocyte (x10 ^9^ cells/L)	Lymhpcyte (x10 ^9^ cells/L)	Neutrophile (x10 ^9^ cells/L)	Platelet (x10 ^9^ cells/L)	C-reactive protein (mg/L)	Aspartate transaminase (IU/L)	Alanine transaminase (IU/L)	Urea (mmol/L)	Creatinine (µmol/L)	D-dimer (µg/ml)
1	*9.1*	1.1*	7.3*	272	70.4*	45*	44	7	0.6	3.2*
2	15.7*	1.7	12.8*	329	4.7	22	10	6.8	0.52	1.8*
3	4.2	1.1*	2.8	144	11.6*	29	12	3.3	0.53	5.9*
4	10.8*	1.8	8.5*	280	18.8*	15	10	6.7	0.36	1.5*
5	9	0.9*	7.8*	308	18.4*	21	9	5.3	0.36	8.1*
6 7 8	8 3.7 14.2*	1.7 1* 2.3	5.7 2.4 10.9*	326 255 162	16.4* 63* 50.2*	27 14 26	23 6 9	4.2 6.6 9	0.38 0.36 0.39	2.4* 4* 0.8*
Median (P25–P75)	9 (5.1 -13.3)	1.4 (1–1.7)	7.5 (3.5–10.3)	276 (185–321)	18.6 (12.8–59.8)	24 (16.5–28.5)	10 (9–20.2)	6.6 (4.4–6.9)	0.3 (0.3–0.5)	2.8 (1.5- 5.4)

Cesarean sections were performed in an isolated operating room. The average birth weight was 3282 ± 191 g. Peritoneal irrigation fluid and amniotic fluid samples were tested for SARS-CoV-2 and all samples were determined to be negative.

## Discussion

There is limited data regarding intraoperative aerosolization of SARS-CoV-2 at the moment. Many medical institutions have different protocols for urgent and elective surgeries. Our institution requires all patients, who are planned to be operated, to be tested for COVID-19 preoperatively both for elective and urgent procedures. Exceptionally urgent procedures may commence before the test result is obtained. The patients in the study were all cases of cesarean section. Since it was possible to postpone other gynecological procedures at least until the patient's test becomes negative, only obstetrical procedures were performed while the patient had active infection.

Personal protective equipments (PPE) should be used by all healthcare staff, who may be in contact with COVID – 19 positive patients, including surgeons, nurses, anesthesiologists and other personnel. Current knowledge is that SARS-CoV-2 is a respiratory virus and possible ways of viral transmission are respiratory droplets, close contact, and fecal-oral route. 8 9 10 11 Airborne transmission has been Although airborne transmission may be considered as an unlikely way for viral transmission, past experiences on the SARS-CoV outbreak shows us aerosol-generating procedures such as tracheal intubation can have the possibility to spread the disease. 12 13 However, there is limited data regarding the management of procedures that can result in aerosolization of Covid-19.

There are several studies that investigated the viral existence in the peritoneal fluid. 7 14 According to our research, there is only one study that demonstrated viral genome in the peritoneal fluid while other studies did not. 15 Absence of the virus may be due to the size of the virus and the pores of the peritoneal membrane. Peritoneal membrane has pores with a maximum diameter of 20–40 nm, in contrast with the diameter of the virus, which is ∼125 nm. 16 SARS-CoV-2 was not detected in the peritoneal fluid according to a case report that investigates SARS-CoV-2 existence in the peritoneal cavity of a non-perforated appendicitis patient. 7 The variation of test methods can play a crucial role in the test results. 20% or even more false negative rates were declared according to the methods of the tests. 17

There are several studies evaluating the viral presence of hepatitis B virus (HBV), human papillomavirus (HPV), and human immunodeficiency virus (HIV) in surgical smoke. All these viruses were detected in the surgical smoke. 18 19 20 21 22 On the other hand, during earlier viral outbreaks, the transmission of other coronaviruses such as SARS-CoV and MERS-CoV through surgical smoke or laparoscopic gas was never confirmed. 23 Limited controversial data regarding the existence of SARS-CoV-2 in the peritoneal fluid, does not seem to create grave concerns for laparoscopic surgery or using electrocautery while operating on COVID-19 positive patients. Surgery of COVID-19 patients should be performed by staff with required PPE and the operating rooms should be managed according to current COVID-19 guidelines. Further studies are needed to investigate the viral presence in the peritoneal fluid to establish more reliable protocols.

Most of the patients were asymptomatic and this may be considered as a limitation, since severe cases concomitantly might have higher viral load and a higher probability of viral presence in the peritoneal fluid. Nevertheless, in the light of our findings, laparoscopic surgery or electrocautery usage should not be avoided due to COVID-19 when indicated and performed under required safety precautions. Our data will contribute to the literature and eventually also help to improve the surgical guidelines for COVID-19 patients.

## Conclusion

SARS-CoV-2 was not detected in the peritoneal fluids or amniotic fluids of all 8 patients with positive nasopharyngeal test results. Apparently, C/S is not a laparoscopic technique and does not provide an ideal example to assess the risks of laparoscopic surgeries. However, the key point is the virus presence in the peritoneal fluid. Although our study is helpful to understand the risk of aerosolization of SARS-CoV-2 during laparoscopy and electrocautery usage, further studies are needed in the field of surgical safety during the COVID-19 pandemic.

## References

[1] Al-Omar K Bakkar S Khasawneh L Donatini G Miccoli P Resuming elective surgery in the time of COVID-19: a safe and comprehensive strategy Updates Surg 2020 72 02 291 295 32495280 10.1007/s13304-020-00822-6 PMC7267759

[2] Bogoch I I Watts A Thomas-Bachli A Huber C Kraemer M UG Khan K Pneumonia of unknown aetiology in Wuhan, China: potential for international spread via commercial air travel J Travel Med 2020 27 02 taaa008 31943059 10.1093/jtm/taaa008 PMC7107534

[3] Chang D Xu H Rebaza A Sharma L Dela Cruz C S Protecting health-care workers from subclinical coronavirus infection Lancet Respir Med 2020 8 03 e13 32061333 10.1016/S2213-2600(20)30066-7 PMC7128440

[4] PanSurg collaborative group Zakka K Erridge S Chidambaram S Electrocautery, Diathermy, and Surgical Energy Devices: Are Surgical Teams at Risk During the COVID-19 Pandemic? Ann Surg 2020 272 03 e257 e262 32541232 10.1097/SLA.0000000000004112 PMC7467049

[5] Mintz Y Arezzo A Boni L The risk of COVID-19 transmission by laparoscopic smoke may be lower than for laparotomy: a narrative review Surg Endosc 2020 34 08 3298 3305 32458289 10.1007/s00464-020-07652-y PMC7250491

[6] Romero-Velez G Pereira X Zenilman A Camacho D SARS-Cov-2 was not found in the peritoneal fluid of an asymptomatic patient undergoing laparoscopic appendectomy Surg Laparosc Endosc Percutan Tech 2020 30 06 e43 e45 32694404 10.1097/SLE.0000000000000837 PMC7682729

[7] Ngaserin S HN Koh F H Ong B C Chew M H COVID-19 not detected in peritoneal fluid: a case of laparoscopic appendicectomy for acute appendicitis in a COVID-19-infected patient Langenbecks Arch Surg 2020 405 03 353 355 32385569 10.1007/s00423-020-01891-2 PMC7210098

[8] Yeo C Kaushal S Yeo D Enteric involvement of coronaviruses: is faecal-oral transmission of SARS-CoV-2 possible? Lancet Gastroenterol Hepatol 2020 5 04 335 337 32087098 10.1016/S2468-1253(20)30048-0 PMC7130008

[9] Rhoads D D Cherian S S Roman K Stempak L M Schmotzer C L Sadri N Comparison of Abbott ID Now, DiaSorin Simplexa, and CDC FDA Emergency Use Authorization Methods for the Detection of SARS-CoV-2 from Nasopharyngeal and Nasal Swabs from Individuals Diagnosed with COVID-19. Vol. 58J Clin Microbiol2020 10.1128/JCM.00760-20 PMC7383529 32303564

[10] Zhang W Du R-H Li B Molecular and serological investigation of 2019-nCoV infected patients: implication of multiple shedding routes Emerg Microbes Infect 2020 9 01 386 389 32065057 10.1080/22221751.2020.1729071 PMC7048229

[11] Moriguchi T Harii N Goto J A first case of meningitis/encephalitis associated with SARS-Coronavirus-2. Vol. 94, International journal of infectious diseases: IJID: official publication of the International Society for Infectious Diseases 2020. p. 55–8. 10.1016/j.ijid.2020.03.062 PMC7195378 32251791

[12] Loeb M McGeer A Henry B SARS among critical care nurses, Toronto Emerg Infect Dis 2004 10 02 251 255 15030692 10.3201/eid1002.030838 PMC3322898

[13] Scales D C Green K Chan A K Illness in intensive care staff after brief exposure to severe acute respiratory syndrome Emerg Infect Dis 2003 9 10 1205 1210 14609453 10.3201/eid0910.030525 PMC3033076

[14] Vudayagiri L Gusz J COVID-19 Positive in Nasopharyngeal Swab but Negative in Peritoneal Fluid: Case Report of Perforated Appendicitis Cureus 2020 12 07 e9412 e9412 32864241 10.7759/cureus.9412 PMC7449633

[15] Coccolini F Tartaglia D Puglisi A SARS-CoV-2 Is Present in Peritoneal Fluid in COVID-19 Patients Ann Surg 2020 272 03 e240 e242 33759843 10.1097/SLA.0000000000004030 PMC7467036

[16] Sadioglu R E Aktar M Gulten E Does SARS-CoV-2 reach peritoneal effluent? Vol. 40, Peritoneal dialysis international: journal of the International Society for Peritoneal Dialysis United States 2020. p.520 1 10.1177/0896860820947331 32998644

[17] Xiao A T Tong Y X Zhang S False negative of RT-PCR and prolonged nucleic acid conversion in COVID-19: Rather than recurrence J Med Virol 2020 92 10 1755 1756 32270882 10.1002/jmv.25855 PMC7262304

[18] Baggish M S Poiesz B J Joret D Williamson P Refai A Presence of human immunodeficiency virus DNA in laser smoke Lasers Surg Med 1991 11 03 197 203 1907345 10.1002/lsm.1900110302

[19] Johnson G K Robinson W S Human immunodeficiency virus-1 (HIV-1) in the vapors of surgical power instruments J Med Virol 1991 33 01 47 50 1901908 10.1002/jmv.1890330110

[20] Neumann K Cavalar M Rody A Friemert L Beyer D A Is surgical plume developing during routine LEEPs contaminated with high-risk HPV? A pilot series of experiments Arch Gynecol Obstet 2018 297 02 421 424 29236173 10.1007/s00404-017-4615-2

[21] Sawchuk W S Weber P J Lowy D R Dzubow L M Infectious papillomavirus in the vapor of warts treated with carbon dioxide laser or electrocoagulation: detection and protection J Am Acad Dermatol 1989 21 01 41 49 2545749 10.1016/s0190-9622(89)70146-8

[22] Kwak H D Kim S-H Seo Y S Song K-J Detecting hepatitis B virus in surgical smoke emitted during laparoscopic surgery Occup Environ Med 2016 73 12 857 863 27484956 10.1136/oemed-2016-103724

[23] Chang L Yan Y Wang L Coronavirus Disease 2019: Coronaviruses and Blood Safety Transfus Med Rev 2020 34 02 75 80 32107119 10.1016/j.tmrv.2020.02.003 PMC7135848

